# P-865. Safety of early suspension or antibiotic de-escalation in febrile neutropenic patients in a Colombian oncologic hospital

**DOI:** 10.1093/ofid/ofaf695.1073

**Published:** 2026-01-11

**Authors:** Maria Jose Lopez, Camilo Buitrago Bahamon, Carlos Fernando Gomez, Jorge Anibal Daza, Paola Omaña, Virginia Abello, Sandra Juliana Galeano

**Affiliations:** Fundacion CTIC, Bogota, Distrito Capital de Bogota, Colombia; Fundacion CTIC, Bogota, Distrito Capital de Bogota, Colombia; Fundacion CTIC, Bogota, Distrito Capital de Bogota, Colombia; Fundacion CTIC, Bogota, Distrito Capital de Bogota, Colombia; Fundacion CTIC, Bogota, Distrito Capital de Bogota, Colombia; Fundacion CTIC, Bogota, Distrito Capital de Bogota, Colombia; Fundacion CTIC, Bogota, Distrito Capital de Bogota, Colombia

## Abstract

**Background:**

There is increasing evidence about the non-inferiority of shortening antibiotic treatment in febrile neutropenic patients, between 3 and 5 days, but there is no strong evidence of de-escalation to class 1-3 betalactam antibiotics. Our objective was to determine if an earlier stop or de-escalation of antibiotic therapy was safe for patients with febrile neutropenia.

**Methods:**

This is an observational retrospective study of an oncological hospital in Bogotá, Colombia, which took place between January of 2023 and December of 2024. We include antimicrobial stewardship program (ASP) evaluated patients with febrile neutropenia diagnosis in whom the antibiotic therapy was suspended or de-escalated according to microbiological isolates. Data was collected from the clinical record.

**Results:**

We included 67 events in 45 patients (Figure and table 1). Piperacillin tazobactam was the main empiric therapy (75% of cases), only 3% of cases had antiMRSA empiric therapy.

In 55% of cases empiric therapy was suspended because of the absence of clinical or microbiological infection (mean 84,6 h IQR 72-96 h).

The main infection was primary bacteremia (62,5%) and *E. coli* was the most isolated microorganism (65,5 %). The empiric therapy was de-escalated to 1st generation cephalosporins (44%), ampicillin-sulbactam (22%) or 2^nd^-3^rd^ generation cephalosporins (22,22%). In 77,6% of the events there was no need for antibiotic restart or re-escalation in the next 7 days. In the 15 events with the antibiotic restarted or escalated (22,4 %), six (40 %) had microbiological infection; the only risk factor for restarting o escalating antibiotics was a lower MASCC (p=0,025).

We had 3 cases of in-hospital mortality (6,5 %), with a mean of 9 days after ASP intervention, none of the cases had an infectious cause (1 oncologic refractoriness, 1 pulmonary edema and 1 CNS bleeding).

**Conclusion:**

We found that ASP interventions (early antibiotic suspension with 48 h negative blood culture preliminary result and 24 hours of apyrexia and de-escalation guided by antimicrobial susceptibility) are safe, but they should be accompanied with close follow-up, and a systematic febrile neutropenia approach for subsequent episodes, mainly in those patients with lower MASCC score at FN diagnosis.

**Disclosures:**

Maria Jose Lopez, Infectious diseases service, Abbvie: Honoraria|Biomerieaux: Advisor/Consultant|Biomerieaux: Honoraria|GSK: Advisor/Consultant|GSK: Honoraria|Knight: Honoraria|Pfizer: Honoraria Paola Omaña, Hematology UFC, Abbvie: Honoraria Virginia Abello, Hematology UFC, Bristol Meyeres Squibb: Grant/Research Support

